# Challenging Methicillin Resistance Detection in Bone and Joint Infections: Focus on the MRSA/SA SSTI® Strategy

**DOI:** 10.3389/fmed.2021.553965

**Published:** 2021-05-17

**Authors:** Marie Titécat, Caroline Loïez, François Demaeght, Jean-Thomas Leclerc, Théo Martin, Hervé Dezèque, Henri Migaud, Eric Senneville

**Affiliations:** ^1^Laboratory of Bacteriology, Institute of Microbiology, Lille University Hospital, Lille, France; ^2^University of Lille, Lille, France; ^3^Northwest Reference Center for Osteoarticular Infections (CRIOAC-G4 Lille-Tourcoing), Lille, France; ^4^University Department of Infectious Diseases, Gustave Dron Hospital, Tourcoing, France; ^5^Orthopaedic Department, Lille University Hospital, Lille, France; ^6^Department of Orthopedic Surgery, Centre Hospitalier Universitaire de Québec-Université Laval, Quebec, QC, Canada

**Keywords:** bone and joint infections, methicillin resistance, PCR, conventionnal culture, Xpert MRSA/SA SSTI®

## Abstract

The genus *Staphylococcus* is the main causative agent of bone and joint infections (BJI) in which outcomes are impacted by both effective surgical and appropriate antimicrobial management. In this context, methicillin resistance (MR) detection is a microbiological challenge to optimize the anti-staphylococcal drug coverage and to secure the surgical procedure. During the last decade, molecular tools have been developed to rapidly detect bacterial-resistant strains in clinical samples. The GeneXpert MRSA/SA SSTI^®^ assay (Cepheid, Sunnyvale, CA, USA) is a real-time PCR method aimed at detecting methicillin-resistant *Staphylococcus aureus* (MRSA) in skin and soft tissues infections. In the literature, this test has been reported to be diverted from its original purpose to be evaluated in surgical samples. Within the current review, we update the GeneXpert MRSA/SA SSTI^®^ assay performance in staphylococcal species determination (i.e., *S. aureus* vs. coagulase-negative species) together with MR genotype detection, when performed in osteoarticular infections.

## Introduction

Bone and joint infections (BJI) encompass a heterogenous group combining native joints and device-associated infections, covering children osteomyelitis, adults' septic arthritis, spondylodiscitis, and prosthetic joint infections (PJI). They require a complex management involving a multidisciplinary approach associating orthopedic surgeons, infectiologists, and microbiologists (1). *Staphylococcus* spp., the main bacterial genus involved in BJI, is reported to be a risk factor associated with inpatient mortality (2). Today, methicillin-resistant coagulase-negative staphylococci (MRCoNS) have become unavoidable in chronic PJI (3, 4) justifying empirical use of glycopeptides (5); while efficiently targeting methicillin-sensitive *Staphylococcus aureus* (MSSA) in native BJI is the key to avoid recurrence and complications (6, 7). In this context, providing rapid bacterial susceptibility results is crucial to guide efficient antimicrobial adaptation in the peri-operative time. Until now, the “gold-standard” method still relies on conventional microbial cultures that require 2–15 days to identify bacterial strains (8) and additional 24–72 h longer to ascertain the antibiotic susceptibility pattern. Moreover, the efficacy of culture methods is partly questionable with a 62.6% sensitivity (9) in PJI cases, and importantly, methicillin resistance (MR) determination may be delicate to rapidly discriminate a heterogenous phenotypical feature and a “borderline” resistance with conventional methods. Hence, there is a need to improve diagnosis methods to reduce the time frame for appropriate antimicrobial management, since total hip and total knee revisions are anticipated to increase by 137 and 601%, respectively, between 2005 and 2030 (10) with 25% attributed to infection (11). During the last decade, the global bacterial resistance burden, including the spread of the virulent community-acquired methicillin-resistant *S. aureus* (CA-MRSA) clone US300 (12), triggered the development of molecular tools aimed at targeting bacterial pathogen DNA and their main resistance determinants. In this attempt, the GeneXpert MRSA/SA SSTI® test (Cepheid, Sunnyvale, CA, USA) was originally designed to detect *S. aureus* (SA) and methicillin-resistant SA (MRSA) directly in clinical samples of skin and soft tissue infections (SSTI). This test was efficiently evaluated in wound and blood culture specimens (13) engaging to divert its former use for osteoarticular applications by distinct clinical units worldwide. Beyond SA and MRSA identification, the assay allows for the specific detection of the genetic support of MR, the *mec*A gene, and interestingly provides the possibility to detect the presence of an MR staphylococcal (MRS) strain from the surgical site, whatever the species. We propose herein to review the performance of the GeneXpert MRSA/SA SSTI® assay for SA, MRSA, and MRCoNS detection and discuss the reliability of such use in several BJI contexts throughout recent articles.

## Basis of the GeneXpert MRSA/SA SSTI® Concept

MR is acquired by horizontal transfer and chromosomal integration of a mobile genetic element designated staphylococcal cassette chromosome mec (SCCmec) (14). The *mec*A gene encodes an alternative penicillin-binding protein (PBP2a), an enzyme responsible for crosslinking the peptidoglycans in the bacterial cell wall, resulting in poor affinity for β-lactams and global resistance to this class of antibiotics (15). The GeneXpert MRSA/SA SSTI® assay is a commercial real-time PCR-based method which relies on the simultaneous detection of three targets: the SA protein A (*spa*) gene, the gene supporting MR (*mec*A), and the SA SCC*mec* chromosomal insertion site which is located at the 3′ end of an unknown function open reading frame, *orfX* (16). All PCR steps (i.e., extraction, amplification, and detection) take place in a single-use cartridge which contains all the reagents necessary for the detection of the three abovementioned bacterial targets together with an internal sample processing control (SPC) (*Bacillus globigii* spores). According to the manufacturer's recommendations, clinical samples may be collected on Copan swabs, discharged in elution buffer, vortexed for 10 s, and then transferred into Xpert MRSA/SA cartridges. The overall analysis is complete in <1 h, and amplification curves are automatically read by the GeneXpert Dx System in terms of MRSA and SA positive or negative, respectively. A comprehensive look may lead to additional interpretations: (i) an isolated amplification of the *spa* gene assesses the presence of MSSA, (ii) the simultaneous detection of the three targets (i.e., *spa, mec*A, and SCC*mec*) attests the presence of MRSA, (iii) a unique amplification of the *mec*A gene can be interpreted as the presence of MRCoNS, (iv) the simultaneous detection of both the *spa* and the *mec*A genes supposes a mixed infection containing both MSSA and MRCoNS strains, and (v) the amplification of the *spa* gene and SCC insertion site without *mec*A signal may be interpreted as an MSSA empty cassette variant. The limits of detection reported by the manufacturer are 150 and 300 CFU/swab for positive SA and MRSA results, respectively.

## Rationale for Evaluating XPERT MRSA/SA SSTI® in BJI

The literature points out only seven publications dealing with the performances of the MRSA/SA SSTI® real-time PCR assay (Cepheid, Sunnyvale, CA) in BJI diagnosis according to distinct protocols. Four out of seven were prospective studies; all but one (17) were led on adult cohorts. These studies were mainly conducted on PJI patients (18–21); unspecified BJI (22); a combination between PJI, spondylodiscitis, and septic arthritis (23); and children suffering from musculoskeletal infections (17). The number of patients included varied from 30 to 213 patients tested for one (21) to at least three distinct samples (18, 19), including joint aspiration, tissue or bone specimens, and prosthetic sonicates in one case (21). Tests of patients diagnosed with staphylococcal BJI were performed either on fresh samples (18, 19, 23) or frozen stored ones (−80°C) (17, 20–22). Biopsies were either directly vortexed (20), grinded, or crushed in saline buffer (17, 22, 23) or even cultured according to beadmill processing (18, 19, 21, 24). In all studies, the liquid phase of the samples was absorbed onto a swab (Copan, Cepheid) from 5 s (23) to 1 min (22) and then discharged in the elution buffer according to the manufacturer's recommendations. Another strategy consisted of directly collecting one Eswab from the periprosthetic tissue during the surgery and vortexing it into a reagent vial from the Xpert kit (21). RT-PCR results obtained from those swabs were compared to identification and resistance patterns reached from corresponding standard (17, 18, 23) and enriched cultures in blood culture bottles (17, 20) in Schaedler (22) or Rosenow broth (19), which were incubated from 5 (17) up to 14 (18, 19, 21–23) or 15 days (20). The main evaluation criteria were the accuracy of MR detection (18–20), the ability of the GeneXpert MRSA-SA SSTI restricted to the identification of SA and MRSA (17, 22), or the latter associated with MRCoNS detection (21, 23).

### Appraising Xpert's Performance in SA/MRSA Detection

SA is the predominant causal pathogen involved in native infections which represent the most frequent clinical form of BJI, accounting for 68% of cases (2). SA also remains the main cause of acute hematogenous BJI in children, representing >90% of methicillin-sensitive strains (25). On the one hand, while most of the studies focus on MRSA, MSSA native BJI are associated with a high rate of treatment failure and further functional sequalae (6), as reported by a three times higher risk of recurrence following vancomycin therapy than observed with β-lactam antibiotics (7). On the other hand, when considering device-associated infections, SA is more likely to be involved in early acute infections (4, 26), while MR phenotype is subject to geographical discrepancies (3). In the USA, SA accounts for 38.6% of surgical site infections following orthopedic surgery, including 38% of MR strains (27), leading to an evaluated cost of $107,264 per case in comparison with $68,053 in case of MSSA (28). In Europe, SA is involved in a similar proportion of PJI (31.9 and 38.7% of total hip and knee arthroplasty infections, respectively) (29), whereas MR proportion follows a downward trend, as confirmed within German (30) and French PJI cohorts (4). When compared to conventional culture (Table 1), the Xpert's test shows attractive performances in SA detection with high sensitivity and specificity ranging from 85.4% (17) to 100% (18, 21, 23) and 91.2% (18) to 100% (21, 22), respectively. Moreover, positive PCR cases related to sterile cultures had to be reconsidered in light of the patient's history (i.e., proven SA infection) supporting the demonstrated sensitivity of the molecular assay. Interestingly, MRSA detection displays an analogous level of efficiency and is associated with a high negative predictive value (NPV 98.9–100%) allowing unambiguous use of β-lactams when SA is detected in osteoarticular samples. Further studies considering both the clinical outcome and the economic impact of such early screening would be of major interest, as suggested by the recent report from AlQahtani et al. (31) in the context of SA bacteremia.

**Table 1:** Comparison of studies evaluating the GeneXpert MRSA/SA SSTI® in BJI diagnosis.

**References**	**Dubouix-Bourandy et al. (23)**	**Titécat et al. (18)**	**Valour et al. (22)**	**Lourtet-Hascoëtt et al. (20)**	**Titécat et al. (19)**	**Sambri et al. (21)**	**Searns et al. (17)**
Study design	Prospective	Prospective	Retrospective	Retrospective	Prospective	Prospective	Retrospective
Aim	MSSA	MR detection	SA/MRSA detection	MR detection	False negative in MR detection	MSSA	SA/MRSA detection
	MRSA					MRSA	
	MRCoNS detection					MRCoNS detection	
Populations studied	BJI (PJI—septic arthritis spondylodiscitis)	Chronic PJI	Adults BJI	PJI	PJI	Chronic PJI	Pediatrics musculoskeletal infections
No. of patients	105	30	76	62	213	70	184
No. of samples	135	104	91	72	NA	70	125
Samples characteristics	Fresh samples	Fresh samples	Frozen samples	Frozen samples	Fresh samples	Peri-operative Eswab	Frozen samples
SA detection in positive samples	18/18	37/37	68/72	NA	NA	11/11	51/59
False positive SA detection	0	0	3^a^	NA	NA	0	2^a^
SA detection performance	Se 100%	Se 100%	Se 94.4%	NA	NA	Se 100%	Se 85.4%
	Sp 97.8%	Sp 91.2%	Sp 100%			Sp 100%	Sp 98%
	PPV 90%	PPV 92.5%	PPV NA			PPV 100%	PPV 93%
	NPV 100%	NPV 100%	NPV NA			NPV 100%	NPV 95%
MSSA detection in positive samples	16/16	28/28	59/63	NA	NA	7/7	41/48
MSSA detection performance	Se 100%	Se 100%	Se 93.6%	NA	NA	Se 100%	Se 85.4
	Sp 98.3%	Sp 100%	Sp 100%			Sp 100%	Sp 98.5%
	PPV 88.9%	PPV 100%	PPV NA			PPV 100%	PPV 95.3%
	NPV 100%	NPV 100%	NPV NA			NPV 100%	NPV 95%
MRSA detection in positive samples	2/2	7/7	9/9	NA	NA	4/4	9/11
MRSA detection performance	Se 100%	Se 100%	Se 100%	NA	NA	Se 100%	Se 81.8
	Sp 100%	Sp 99%	Sp 100%			Sp 100%	Sp 100%
	PPV 100%	PPV 87.5%	PPV NA			PPV 100%	PPV 100%
	NPV 100%	NPV 100%	NPV NA			NPV 100%	NPV 98.9%
MRCoN detection	19/19	13/17	NA	9/25	NA	14/16	NA
MRCoN detection performance	Se 100%	Se 76.5%	NA	Se 36%	NA	Se 87.5%	NA
	Sp 95.3%	Sp 95.4%		Sp 98%		Sp 100%	
	PPV 85.2%	PPV 76.5%		PPV 90%		PPV 100%	
	NPV 100%	NPV 95.4%		NPV 74%		NPV 96.4%	
False positive MR detection	4	5^a^	0	1^a^	NA	0	NA
False negative MR detection	0	4 (1 MRSA + 3 MRCoN)	0	16 MRCoN	6 (1 MRSA + 5 MRCoN)	2 MRCoN	2 MRSA
MR detection Xpert's overall performance	Se 100%	Se100%	Se 94.4%	Se 36%	Se 75%	Se 90%	Se 81.8%
	Sp 96.3%	Sp 91.2%	Sp100%	Sp 98%	Sp 93%	Sp 100%	Sp 100%
	PPV 86.2%	PPV 92.5%	PPV NA	PPV 90%	PPV 58%	PPV 100%	PPV 100%
	NPV 100%	NPV 100%	NPV NA	NPV 74%	NPV 97%	NPV 96%	NPV 98.9%

*NA, not available*.

a *Re-interpreted as “true positive” according to patient's history or other results of sample's culture*.

### Appraising Xpert's Performance in MRCoNS Detection

MRCoNS have gained global attention in recent years and are responsible for a large proportion of PJI in European countries (3). While CoNS are less pathogenic than SA, they are able to adhere and colonize orthopedic devices by producing a biofilm. This structure encloses low amounts of slow-growing bacteria and causes delayed infections characterized by subtle symptoms, making their clinical and microbiological diagnosis challenging. Moreover, these infections involving a large proportion of MRCoNS (3, 4) justify empirical use of vancomycin in combination with a broad-spectrum β-lactam (5, 32). High dosage regimens are required to ensure an effective bone diffusion of the antibiotics, but this strategy leads to a significant rate of adverse effect (33) that could be spared by directly detecting MRCoNS in the surgical site. Xpert's accuracy was addressed in this aim by targeting the *mec*A gene in solid and liquid osteoarticular samples (18–21, 23). The test was first validated by Dubouix-Bourandy et al. (23) on 25 samples isolated from various BJI conditions with sensitivity, specificity, positive predictive value (PPV), and NPV values of 100, 95.3, 85.2, and 100%, respectively. These results were further corroborated on chronic PJI patients (18, 22) with an acceptable sensitivity (76.5 and 87%) and a high NPV (95.4 and 96.4%). Nevertheless, Lourtet-Hascoëtt et al. (20) questioned these performances due to a significant number of false negative results (16/25) and highlighted inconsistent sensitivity and NPV, respectively, 36 and 74%. These 16 samples were all frozen and originated from 14 periprosthetic tissues and 2 articular fluids. In contrast, the three other series were performed on fresh tissue samples or extemporaneous periprosthetic tissue swabs directly processed in the peri-operative time. Furthermore, when applied to a larger prospective cohort of 213 patients and 639 osteoarticular specimens (19) and considering Xpert's performance from a patient point of view, only 6 out of 213 patients (2.8%) were misdiagnosed, among whom 5 were infected by MRCoNS strains. It is worth noting that rapid and appropriate antimicrobial adaptation could be delivered to 194 out of 213 patients (91%).

## Few Restrictions of the Test

Reaching 100% accuracy for a diagnostic tool is unrealistic in routine practice, especially when the microbiologist must deal with all the bacterial subtleties related to BJI contexts. Although we highlighted the good performances of the MRSA/SA SSTI above, the limits of this test in terms of false positive and false negative results must be discussed, owing to their potential impact on the antimicrobial strategy. Indeed, a false positive assay may lead to an inappropriate prescription of a broad-spectrum molecule with individual and ecological side effects, and a loss of opportunity to heal in case of MSSA infection. Interestingly, false positive tests have rarely been observed in the seven studies (Table 1) including 5 and 10 reported cases of SA and MR detection, respectively. These cases were mainly related to bacterial DNA detection in patients with an anteriority of infection or who had prior antimicrobial therapy leading to sterile cultures. Finally, these false positive cases could be re-interpreted as a true positive one, highlighting the limit of the gold standard used for the evaluation of this molecular assay. Secondly, worse than a false positive result, a false negative one may jeopardize surgery and the implanted device, implying revision procedure and prolonged hospital cost and length of stay associated with a morbidity increase. These false negative cases were more frequent in MRCoNS infections (18–20). This slightly lower accuracy comparative to SA infections must be interpreted according to the assay's content (i.e., specific probes targeting SA and MRSA) and also physiopathological considerations relative to acute SA infections involving high inoculum of bacteria and chronic MRCoNS infections involving low amounts of biofilm embedded bacteria, not evenly distributed at the implant's surface. Consequently, in a chronic context, the number of samples analyzed should not be restricted to a single one for correct interpretation. Multiplying samples and PCR tests may entail a financial burden for clinical laboratories that should counterbalance the economic consequences of misdiagnosis for healthcare settings. So far, the number and the kind of osteoarticular specimens required for a contributive analysis have to be defined. Moreover, regarding the software's interpretation algorithm, the latter is configured for MRSA/SA detection in skin and soft tissue samples with positivity reports related to abundant bacterial load resulting in low PCR Ct values. Consequently, these criteria cannot be extrapolated to osteoarticular or MRCoNS infection contexts. Accordingly, in BJI indication, the microbiologist's appraisal is required to interpret amplification curves, and particularly late Ct values of the *mec*A gene, in order to not miss a positive sample (18, 19). Finally, other false negative results were also reported in cases of staphylococcal small colonies variants or polymicrobial infections, without any obvious explanations yet.

## Relevance of the MRSA/SA SSTI® in Routine Practice?

Although conventional culture is a perfectible gold standard, it remains the key method to document the infection and to provide an exhaustive antibiogram. The use of blood culture bottles has significantly reduced the time for micro-organism detection with a sensitivity increased to 87% (9) allowing, in the most favorable conditions, phenotypical antimicrobial data in 48 h. The aim of the MRSA/SA SSTI® assay is to reduce this time frame to a couple of hours by targeting the main resistance determinant, i.e., the *mec*A gene, which is decisive for empirical antimicrobial therapy adaptation. In contrast to the 16S rRNA PCR (34, 35) or other home-designed multiplex PCR panels (36), the Xpert's test targets a single bacterial genus but delivers results in 72 min (23) vs. 2 days and half a day, respectively. Xpert is also easily implementable in the routine workflow of a clinical laboratory, with a hand-on time of 2 min (23), and does not involve the use of complex molecular facilities nor dedicated technical supports. Alternatively, an equivalent concept is proposed by the automated multiplex PCR Unyvero i60 ITI (Curetis, Holzgerlingen, Germany). This cartridge system targets 52 pathogens at the genus level, among which are 15 bacteria and yeasts at the species level, and 19 antimicrobial resistance markers delivering available results in 5 h. This assay has been evaluated in PJI diagnosis in three studies (37–39), although MR detection accuracy was only addressed by Malandain et al. (38). Unfortunately, when tested on culture-positive samples, no more than 35% of *bla*_*mecA*_ gene amplifications were detected.

Collectively, these data fully support the relevance of the Xpert's assay in osteoarticular infections for rapid antimicrobial adaptation in the peri-operative time along with its applicability in routine practice. One may assume that this strategy of early and accurate diagnosis is cost-effective; however, this point has to be fully demonstrated for acute and chronic indications, respectively. Nowadays, the room for such new molecular methods in the diagnostic strategy of BJI remains to be clearly defined in clinical and microbiological guidelines.

## Author Contributions

MT drafted the manuscript. ES, CL, HM, J-TL, FD, TM, and HD reviewed the article and provided critical insights. All authors contributed to the article and approved the submitted version.

## Conflict of Interest

ES declares personal honoraria from Cepheid for speaker activity. The remaining authors declare that the research was conducted in the absence of any commercial or financial relationships that could be construed as a potential conflict of interest.
